# A New Process Model for Relationship‐Centred Shared Decision‐Making in Physical Medicine and Rehabilitation Settings

**DOI:** 10.1111/hex.14162

**Published:** 2024-08-14

**Authors:** Christina Papadimitriou, Marla L. Clayman, Trudy Mallinson, Jennifer A. Weaver, Ann Guernon, Albert J. Meehan, Trisha Kot, Paige Ford, Roger Ideishi, Christina Prather, Philip van der Wees

**Affiliations:** ^1^ School of Health Sciences Oakland University Rochester Michigan USA; ^2^ Department of Population and Quantitative Health Sciences Edith Nourse Rogers Memorial Veterans Hospital, UMass Chan Medical School Bedford Massachusetts USA; ^3^ Department of Clinical Research and Leadership George Washington University Washington DC USA; ^4^ Department of Occupational Therapy Colorado State University Fort Collins Colorado USA; ^5^ Department of Speech‐Language Pathology Lewis University Romeoville Illinois USA; ^6^ Department of Sociology, Anthropology, Criminal Justice, and Social Work, College of Arts & Sciences Oakland University Rochester Michigan USA; ^7^ Care Partner Collaborator; ^8^ Department of Occupational Therapy George Washington University Washington DC USA; ^9^ Division of Geriatrics and Palliative Medicine George Washington University Washington DC USA

**Keywords:** physical rehabilitation, relationship‐centred care, shared decision‐making

## Abstract

**Introduction:**

We present a relationship‐centred shared‐decision‐making (RCSDM) process model to explicate factors that shape decision‐making processes during physical medicine and rehabilitation (PMR) encounters among patients, their care partners and practitioners. Existing shared decision‐making (SDM) models fall short in addressing the everyday decisions routinely made regarding persons with chronic disabilities who require high levels of support, their care partners and rehabilitation practitioners. In PMR, these everyday decisions are small scale, immediate and in service to a larger therapeutic goal. They can be thought of as micro‐decisions and involve multiple practitioners, care partners and patients. How micro‐decisions are made in this context is contingent on multiple roles and relationships among these relevant parties. Our model centres on micro‐decisions among patients, their care partners and practitioners based on our disorders of consciousness (DoC) research.

**Methods:**

To develop our model, we examined peer‐reviewed literature in SDM in PMR, chronic disability and person‐centeredness; formed collaborations and co‐created our constructs with rehabilitation practitioners and with care partners who have lived experience of caring for persons with DoC; analysed emerging empirical data and vetted early versions with expert scientific and clinical audiences. Our model builds from the core tenets of relational autonomy, and scholarship and activism of disability advocates.

**Findings:**

Our model conceptualizes four non‐hierarchical levels of analysis to understand the process of micro‐decision‐making in chronic disability and medical rehabilitation: social forces (historical and sociological); roles and relationships (multiple and intersecting); relational dimensions (interactional and contextual) and micro‐decision moments (initiation, response and closure).

**Discussion:**

Relationships among patients, their care partners and practitioners are the intersubjective milieu within which decisions are made. Our conceptual model explicates the process of micro‐decision‐making in PMR.

**Patient or Public Contribution:**

Care partners (or caregivers) and rehabilitation practitioners are active members of our team. We work together to develop research projects, collect, analyse and disseminate data. The conceptual model we present in this manuscript was co‐created—input from care partners and practitioners on previously collected data became the impetus to develop the RCSDM process model and share co‐authorship in this manuscript.

## 1 Introduction

Shared decision‐making (SDM) is a collaborative process in which relevant parties (e.g., patients and healthcare practitioners) make decisions informed by scientific evidence and personal values. SDM requires elicitation and sharing of patient preferences and values to inform options *best* suited to the patient, particularly when more than one evidence‐based equivalent option is available. SDM in physical medicine and rehabilitation (PMR) may differ from other settings because many of the options may not have clear evidentiary support, be about everyday decisions rather than major medical procedures and involve practitioners from multiple disciplines, multiple care partners and the patient. How SDM happens in PMR settings is not well articulated in the literature. (Throughout this manuscript, we refer to persons or patients with ‘chronic disability who require high levels of support’ rather than with ‘severe chronic disability’ because our care partner collaborators tell us they prefer language that focuses on the need for support rather than on a categorical, but essentially unknowable, degree of disability. The patient is the person served by healthcare practitioners in health settings with various insurance coverage. The patient or person is also someone's loved one. Therefore, choice of words has to do with context: in healthcare settings, it's patient; for the care partner, it's loved one; for research, it can be person served or person. We use the term ‘care partner’ to connote the mutuality and reciprocity that may exist between the caregiver and care recipient. The care partner collaborators in our team are in support of this term.) 

This paper proposes a new process model of relationship‐centred shared decision‐making (RCSDM) for PMR and specifically for persons with high support needs. When persons cannot advocate for themselves due to significant cognitive and/or communication disabilities—such as when experiencing disordered states of consciousness (DoC), dementia or developmental or intellectual disabilities—care partners assume much of the responsibility for making decisions. Nonetheless, persons with high support needs remain an important part of decision‐making processes. Care partners are caregivers such as family members or friends who speak for both themselves and their loved ones during decision‐making processes with practitioners [1, 2, 3, 4]. In that sense, they are partners in caring for the loved one, providing daily care and executive/legal decision‐making. During SDM, care partners and practitioners may acknowledge, ask questions of or affirm a loved one's preferences or feelings, even though patients may be unable to self‐report or advocate for themselves [5, 6, 7, 8].

Relationship‐centred care is not a new concept, although it is not usually considered in the context of SDM (for a notable exception, see Beach [9]). When relationship‐centred care and SDM are discussed together, the physician–patient relationship is the primary focus [10]. However, in rehabilitation settings, care is coordinated and delivered by teams of practitioners, several of whom may be present during a treatment session with the care partner and patient. A focus on the physician–patient relationship is not particularly informative in this clinical context. Yet, care partners and rehabilitation practitioners struggle to collaborate in SDM: care partners do not always feel listened to, and practitioners have difficulty addressing the ambiguity and uncertainty of patients' complex diagnoses while treating patients, and thus, collaborating with care partners can be challenging [2, 3, 11]. In presenting our RCSDM process model, we provide one example from our team's empirical research and clinical experiences with persons in DoC and their care partners. We believe that our work may be applied more generally to persons who require high levels of support due to cognitive and communication disabilities, but this remains an empirical question. Our model explicitly acknowledges and includes multiple relevant parties who may be engaged in clinical encounters, such as multiple care partners (the legal surrogate and other unpaid or paid caregivers) and multiple practitioners (disciplines including, but not limited to, physical and occupational therapy, speech‐language pathology, music therapy and nursing). A key implication of our model is that persons with chronic disabilities who require high levels of support need to be recognized and appreciated as relational agents [12, 13, 14, 15, 16, 17]. We envision this as a ‘living model’, a tool that is valuable in its present form, and one that we and others will refine as more data are collected and analysed and as the descriptive and conceptual language around relationality continues to improve.

## 2 The Need for a New Model and Our Design Approach

The impetus to develop this model arose when our cross‐disciplinary team analysed interview data from rehabilitation practitioners and care partners who care for persons with DoC after traumatic brain injury and from observational data from clinical encounters among care partners, rehabilitation practitioners and patients with DoC [18, 19, 20]. In our data, care partners talked about the challenges of not feeling listened to by practitioners [2, 3] and therefore not always included in the decision‐making for their loved ones. Rehabilitation practitioners experience ambiguity and uncertainty when they treat persons in DoC, and though they recognize the importance of including care partners in their treatments, they are challenged to do so [11]. Further, we noticed that decision‐making did not follow the traditional SDM process of deliberation that the literature describes. We applied the concept of everyday micro‐decisions [21] to our analyses and began explicating its particular features in our data. We searched the existing systematic literature reviews of SDM models and tools [8, 22, 23] to learn how collaborative decision‐making occurs among care partners and practitioners from multiple disciplines. We wanted to understand how everyday micro‐decisions were made in healthcare settings where multiple practitioners encounter patients and their care partners frequently as part of their care plans. We did not limit ourselves to rehabilitation settings, rather we searched the general medical and rehabilitation literature including settings such as emergency departments, neonatal intensive care and dementia care. We found that there are no models built on the concept of micro‐decisions that centre on the relationships among patients, their care partners and practitioners in medical rehabilitation. One model on trust [24] in intensive care captured our interest because it articulated dimensions of influence that aligned with our relational conceptual underpinnings (see below). We therefore began building our model on our data analyses and literature reviews by engaging with rehabilitation practitioners and care partners who shared their lived experiences and reflected on versions of the model. That early work was shared with rehabilitation practitioners as well as SDM scholars [1, 25] and received positive feedback.

For this PMR environment, we find several shortcomings in how SDM is usually described. First, while SDM is consistently referred to as a process, some widely cited models primarily reflect on practitioner competencies [6]. In other words, they tend to be practitioner centric. Second, SDM models fall short in addressing the types of decisions that occur during PMR therapy sessions [7, 8].

SDM models have centred on outpatient visits with patient‐provider decisions for which there is ample evidence for deciding among clearly defined options with known outcomes. In those settings, while decision‐making may be considered a process, the decision itself is often a one‐time, irrevocable decision (e.g., deciding between lumpectomy and radiation vs. mastectomy for early‐stage breast cancer). Many of these decisions are not enacted in the encounter itself, allowing for reflection and discussion (see Table 1 for a summary of key points). Recent work has expanded to look at SDM in other settings, including inpatient hospital care [26, 27] and home health [28], as well as decisions for which there is little evidence to guide patients and practitioners in decision‐making. These are closer to rehabilitation environments in that they describe decisions and care processes that take place over time, with multiple types of practitioners, concurrently or sequentially. However, care partners are generally not included in the SDM conceptual models, and the decisions are still primarily enacted at a later time [21, 29].

**Table 1:** Some key differences between office‐based and PMR therapy encounters as they apply to SDM.

Office‐based encounters	PMR therapy encounters
Happen occasionally	Happen several times a day
Typically have one practitioner	May involve several practitioners
Practitioners are typically physicians, nurse practitioners and physician assistants	Practitioners are a wide variety of practitioners (e.g., music/occupational/physical/speech/nursing)
Decision‐making is discussion‐based; involves deliberation	Micro‐decision‐making relies on verbal and physical actions and responses
Decisions are enacted after encounters	Micro‐decisions are enacted during encounters
Evidence‐based to inform choices includes randomized controlled trials	Variety of evidentiary support

### 2.1 The Role of the Person With DoC in SDM

**We argue that persons who require high levels of support and cannot independently communicate their needs remain a vital part of the decision‐making process because their values, interests, responses and reactions to treatment are what care partners and practitioners appraise and accommodate when making decisions** (for similar points, see De Hass and colleagues [30, 31]). Although it is care partners who predominantly interact with practitioners, the patient is physically present during encounters and may show that they are aware of the presence of others and provide responses to sensory stimuli such as familiar voices, familiar faces or touch, such as a hand on their forearm. In addition, since the science remains unclear as to how much patients understand, best practice in DoC care is to interact as if the person understands what is being communicated. The care partner bases their decisions on their prior relationship with their loved one (where relevant, including a pre‐injury or pre‐disease relationship) as well as current caregiving experiences. For instance, they are likely to notice their loved one's bodily expressions and interpret them in ways imbued with meaning. Without care partners' noticing, patients' bodily and vocal expressions not only might go unnoticed by practitioners but may be perceived as not substantive or relevant [32]. Rehabilitation practitioners attempt to identify meaningful responses to sensory stimuli through evidence‐informed assessments and clinical observations. Nonetheless, they often find themselves struggling to make sense of patients' neurobehavioural responses [10, 33]. Therefore, **the encounter is the environment wherein these relevant parties interact and decisions are made**. We contend that extant SDM models fail to adequately account for this relational dimension.

### 2.2 A Note on Team Positionality

The development of our model engaged a multidisciplinary team comprising an SDM expert (M.L.C.), three occupational therapists (T.M., J.A.W. and R.I.), a speech language pathologist (A.G.), a physical therapist (P.v.d.W.), two family care partners (T.K. and P.F.), two sociologists (C.P. and A.J.M.), a Rasch measurement expert (T.M.) and a physician (C.Pr.). Combined, the team has expertise in rehabilitation intervention of persons with DoC and their families, psychometrics, health services research, translational research, community‐engaged research and person‐centred care practices. As a team, we are sensitive to the value of giving voice to often‐ignored perspectives and collaborating with family care partners and rehabilitation practitioners to understand how decisions are made [34].

## 3 The Need to Focus on the SDM Process: Introducing the Concept of Micro‐Decisions

One important aspect of SDM models is deliberation, that is, thoughtful discussion that includes patient goals and preferences along with clinical expertise. However, what exactly makes an interaction fulfill the definition of ‘deliberative’ is unclear. Clayman et al. [32] provide a useful model for thinking about SDM in rehabilitation. Conceptually, the Clayman et al. model expands the thinking about SDM in several ways: it is more inclusive of the temporal aspects of decision‐making (e.g., the work done by patients before and after visits); recognizes that any individual decision or medical encounter is just one brief part of an entire illness narrative and shifts the scope of measurement to include the entirety of the decision process, rather than solely focussing on a single clinical encounter. The model is both narrative (i.e., intentional meaning‐making) and focussed on the person in the context of their life and relationships. These two aspects (narrative and personhood) are integral to decision‐making because the former is how people make sense and meaning out of events while the latter acknowledges that relationships, values and non‐medical concerns are also essential to how people make decisions. That is, people are not solely rational actors who make decisions entirely logically or entirely on their own. Our RCSDM process model builds on this conceptual work.

However, as described above, nearly all extant models of SDM, including Clayman's, presume that a decision to be made centres on a conversation between a practitioner and a patient (or care partner). As we will describe below, these models do not take into account the immediacy and physicality of much of what occurs in rehabilitation encounters. The very act of a rehabilitation therapy encounter may contain decisions and immediate changes in treatment based on patients' reactions and care partners' behaviours or preferences.

In traditional SDM, deliberation takes the form of a cognitive process among competent adults in a clinical outpatient encounter for which more than one option is available, and each option has clear evidence to delineate likelihoods of possible health outcomes [35]. By contrast, decisions often made in rehabilitation practice can be thought of as **micro‐decisions**, that is, small‐scale decisions made, enacted and evaluated in the moment in service to a larger treatment or therapeutic goal [36].

What differentiates these micro‐decisions from other clinical decision‐making is threefold: (1) they are in the moment and immediate; (2) they are generally done with healthcare practitioners who are not physicians and may involve multiple relevant parties (e.g., several rehabilitation practitioners, care partners and patient); (3) they have an intimacy that derives from the necessity of physically close, intimate and frequent interactions between patient, care partner and practitioners [36]. During rehabilitation encounters, multiple micro‐decisions take place, most of them without explicit deliberation, around decisions for which there is no clear evidence, and for which the outcome may be unknown. These decisions involve navigating the complexities of mundane, everyday decisions that are nonetheless consequential to the relational dimensions and processes of clinical exchanges. Below is an example of a micro‐decision that will elucidate our model.

**Example from our PMR research in disorders of consciousness (DoC)**

Micro‐decision: mint or cologne?

In an acute care hospital, two practitioners (RPs), Bettina (Physical Therapist RP2) and Lucy (Occupational Therapist RP4), are in the room of patient, Ken. They are considering administering the odour of mint to document whether it elicits a response that can be used to reliably gauge Ken's reaction to scent. The use of mint is part of the professional and scientific toolbox that practitioners commonly use during clinical assessments in DoC, and something that these practitioners have used before with Ken with some success. Ken's mom and care partner, Clarisse (CareP), proposes cranberry juice as an alternative option. The care partner has presented the practitioners with a micro‐decision: *Use cranberry juice, or continue with the mint to gauge Ken's reaction to scent*? Bettina does not take up Clarisse's offer. Clarisse then points to Ken's favourite cologne and suggests to Bettina and Lucy that they use it instead. The care partner has presented the practitioners with another opportunity for micro‐decision: *Use the cologne, or continue with the mint to gauge Ken's reaction to scent*? The RPs take up Clarisse's offer.

As this encounter unfolds, the micro‐decision does not allow for, nor would it necessarily be appropriate to have, extended deliberation about values and preferences. Nor is there clear evidentiary support for using one fragrance over another to elicit responses in patients with DoC. Yet how this micro‐decision is attended to matters for the relationships among these participants, and importantly for the patient's trajectory of care since eye‐tracking since localized responses to stimuli are important determinants of improvement.

## 4 Conceptual Underpinnings

Our model builds from the core tenets of relational autonomy [12, 13, 14, 15, 16], research in person centeredness in medical rehabilitation [37, 38, 39], scholarship in healthcare SDM and scholarship and activism of disability advocates [40, 41]. We bring a hermeneutic approach to our understanding of human behaviour and relations [42] to emphasize that connection is foundational to human existence [43, 44, 45] and that individuals always exist within webs of meanings and relationships [11, 24, 42, 46, 47]. Humans are emotional and social beings who make meaning as part of everyday interactions with each other and with social institutions. Humans exist in cultural milieux that affect our behaviours, decisions and perceptions (of ourselves and others). In other words, we are relational beings [12, 13, 14, 15, 16]—our relationships with others, with organizations and with cultural norms are relevant in the construction of our sense of self, our habits and, therefore, our preferences, choices and actions (including what we take to be rational). This is not a radical view, but it challenges the individualistic and patriarchal view of autonomy often dominating healthcare, one which presumes a solitary patient making decisions in isolation, independent of social and emotional contexts, devoid of affinities with others and who has sole decisional responsibility. Our model is framed by these perspectives because they best capture the social and familial relations relevant to a person's life and the micro‐decisions enacted in PMR encounters. Our model centres the relationships among care partners‐patients‐practitioners because they constitute the environment in which decision‐making exchanges occur in physical rehabilitation for persons who need high levels of support. Whether this model can be applied to other similar settings is an empirical question beyond the scope of this paper.

## 5 RCSDM Process Model

**Figure 1:**
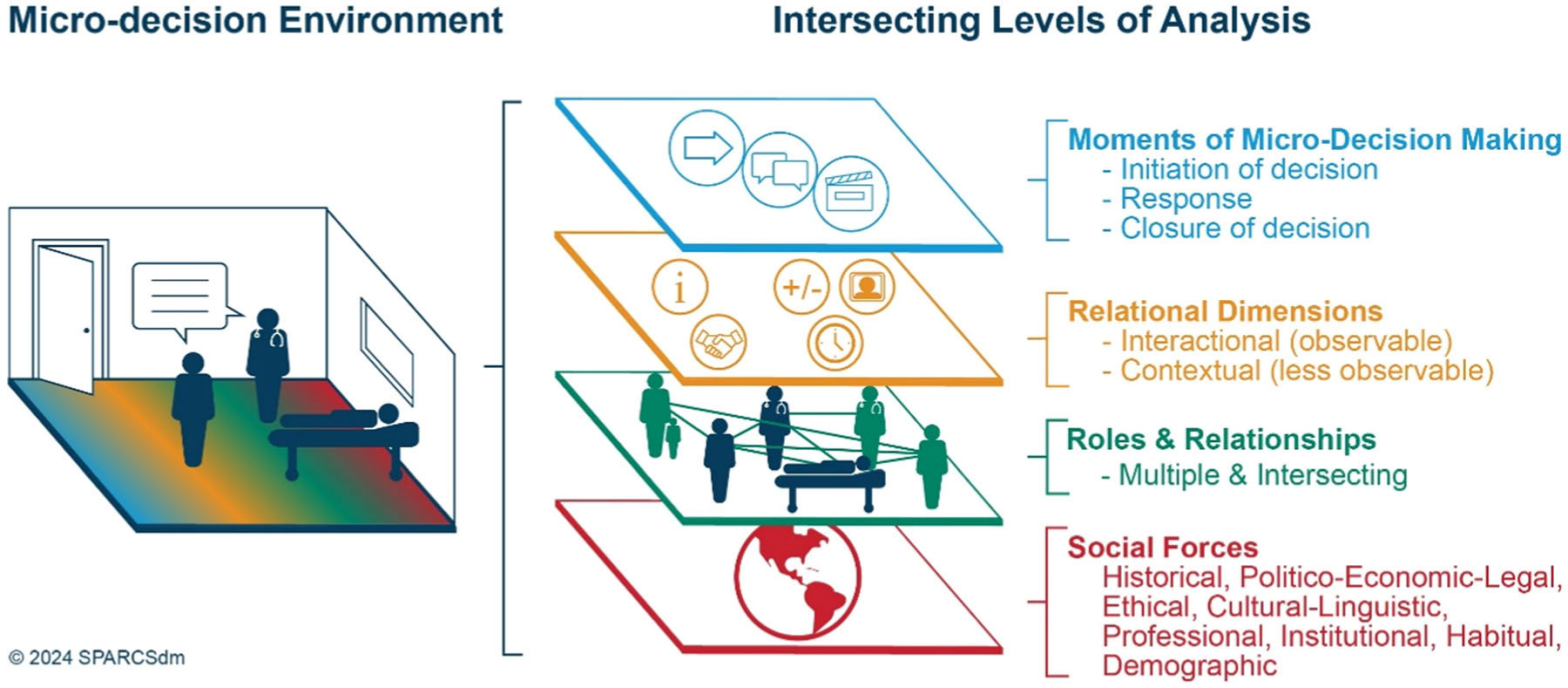
Schematic representation of the relationship‐centred shared decision‐making process model.

The RCSDM process model focuses on explicating factors that shape decision‐making processes during rehabilitation encounters among patients, their care partners and practitioners. The micro‐decision is the environment in which we can observe how this decision‐making takes place [36]. To make explicit what occurs in the micro‐decision environment, we distinguish four analytic levels that intersect and together form the intersubjective environment of decision‐making. To explicate them, we separate them, but the ordering of these levels in Figure 1 is not intended to convey a hierarchical relationship or importance.

SDM is a dynamic process that is influenced by and that influences relationships that, in turn, are always already affected by social forces such as institutional and professional norms. In this environment, there is scientific and epistemic uncertainty regarding the accurate detection of consciousness states and effective treatments [11, 48]. There may be differing interpretations of patient behaviours based on previous experiences with the person, as well as practitioners' disciplinary training.

### 5.1 Level 1: The Three Moments of Micro‐Decisions

The experience of making micro‐decisions is complex, nuanced and situational. Micro‐decisions can be identified through observation and involve what we refer to as the three moments of micro‐decision‐making: initiation, response and closure [25]. In the ‘mint or cologne?’ example, the care partner (Clarisse, CareP) offers her son's favourite cologne as a possible way forward in the rehabilitation encounter, thereby **initiating** a micro‐decision: *Will the practitioner use the cologne or continue with their commonly‐used approach with mint (or other items they often employ) to gauge the patient's reaction to scen*t? This is not a simple binary (yes/no) decision. It is a fluid, dynamic and interactional process. How the initiation is received by the RPs will influence their response and thereby the extent to which they will collaborate in this therapeutic moment. Here, we provide an analysis of how this decision happened. In a separate paper, under preparation, we explicate these three moments of micro‐decisions in more detail.

The mint or cologne micro‐decision occurred right after the care partner initiated another micro‐decision that was not accepted by the RPs. The rehabilitation practitioners (RPs Bettina and Lucy) were discussing possible options for testing the patient's (Ken) reaction to scent commonly used for this purpose. Clarisse (CareP) proposed cranberry juice as a possible option for them to use and offered to retrieve it from the counter. In doing so, the care partner's initiation created an interactional opportunity for a micro‐decision to occur: *Do the practitioners accept using cranberry juice or continue with using mint?*

Bettina's **response** rejects Clarisse's offer but with an apology, stating that in a previous session Ken ‘shrugged [the mint] off’. This prior positive reaction to mint and the fact that mint is part of the professional toolbox practitioners commonly use during clinical assessments of DoC form this micro‐decision environment which may therefore influence it. However, in this rejection of Clarisse's initiation, Bettina also expresses some uncertainty about the mint's possible success stating ‘we're just trying anything and everything’, which may represent an opportunity, an openness, to do something new. That is when Clarisse **initiates** a second micro‐decision when she shows Ken's cologne: *Do the practitioners use the cologne or continue with the mint to gauge the patient's reaction to scent?* Both RPs accept and incorporate this initiation.

Table 2 shows the text of the mint‐cologne exchange in italics. It has been adapted from our data transcript [19]. Nonverbal actions are in double parentheses.

**Table 2:** The mint or cologne micro‐decision exchange.

CareP: *there's some juice over there, cranberry juice* ((moves to retrieve from counter, RP4 leaves the room))
RP2: *I think. I don't. I'm trying, I'm sorry. I think I'm going to try this first, see if the smell of like the minty does anything? That one day he kinda like shrugged it off. We're just trying anything and everything* ((RP4 returns))
CareP: *Your cologne again* ((looking at Ken, and showing bottle to RPs))
RP2/RP4: *Oh, the cologne!* ((said together))
RP2: ((nods affirmatively and looks at CareP and then patient))
RP4: *Here it comes*
RP4: ((said to CareP)) *Yeah, now try the cologne on this side ((points to the upper left)) see if he'll look at it up there*
((The RPs are positioned in front of Ken to keep him stable. CareP is behind Ken and moves the cologne bottle into Ken's view and sprays the scent. Ken turns his eyes and looks toward CareP and the cologne bottle))
RP4: *Yeah!* ((excited voice))
RP2: *Yeah, cool!* ((excited voice))
CareP: *He liked what he saw?*
RP2: *Yeah, he was looking at it and his eyes went from this to there*

Abbreviations: CareP = Clarisse; Patient = Ken; RP2 = Bettina; RP4 = Lucy.

The practitioners **respond** by accepting this second initiation. This is noted by both RPs immediate verbal and affective response to the care partner's suggestion ‘Oh the cologne’ alluding to a possible familiarity with the cologne and the meaning it has for this family. The acceptance of this initiation is further shown by how the RPs incorporate the care partner into the actual process (‘now try the cologne on this side’). Care partner and RPs have had previous interactions, and this current encounter is therefore potentially influenced by past ones and by their relationships. This micro‐decision concludes when CareP asks the RPs ‘he liked what he saw?’ and RP2 provides a positive appraisal of Ken's response (eye tracking), thus marking a **closure** to this micro‐decision. Our research and clinical expertise in DoC suggest that these micro‐decisions are a more routine occurrence in rehabilitation encounters and an important part of the clinical reasoning skills practitioners exhibit during encounters [11, 48].

### 5.2 Level 2: Relational Dimensions of Decisions

When practitioners, care partners and patients interact during clinical encounters, they bring with them experiences, beliefs, information and skills that shape their behaviours and interactions and the micro‐decisions that take place within them. In our model, we identify five relational dimensions that may influence SDM during micro‐decision‐making that we categorize as either interactional or contextual.

Within the **Interactional** category, dimensions are observable actions that relevant parties may exhibit during an encounter, specifically information exchange (IE) and collaboration skills (CSs). Within the **Contextual** category, dimensions are less observable but analytically accessible. They include stakeholder experiences and beliefs, time of encounter regarding a persons' disability trajectory and perceived value and meaning of decision [48].

We use the term dimensions because it denotes variation. Micro‐decisions are instances in which these dimensions are present. We explain each dimension and how they influence the process of SDM. These dimensions may co‐occur and do not have a hierarchical order.

#### 5.2.1 Interactional

Interactional dimensions are **observable** verbal and nonverbal actions that relevant parties exhibit during encounters.

##### Information exchange

Communicating information about the patient's needs, status, behaviour, treatment plan and so on includes *what* is communicated, the *quality* of the information, its *timeliness* or *relevance* whether it was *clearly* communicated, culturally *competent,* and *person‐*centred. How that information is communicated involves skills underlying relevant capacities (e.g., training, expertise) [49] and what the environmental demands call for in the moment [50].

In Table 3, an example of IE is enacted by the care partner as a person‐centred knowledge claim [51] (‘your cologne again’) in a timely and relevant moment right after RP2 stated ‘we try anything and everything’. The practitioners respond and appraise this information (‘yeah cool’), which results in trying out the cologne.

**Table 3:** Description of collaboration and information exchange skills in micro‐decision‐making.

Description of micro‐decision exchange	Analysis of behaviours displayed: IE	Analysis of behaviours displayed: CSs
**Clarisse** looks at **Ken**: *Your cologne again*.	**CareP**: Timely and comprehensible Pt preference communicated	
**Clarisse** shows cologne to **Ken, Bettina and Lucy**	**Initiates** to use the cologne	**RP**: Active listening via eye contact, body language, smile and tone of voice; acknowledgement
**Bettina and Lucy:** *Oh, the cologne!* ((looking at Ken, and showing bottle to other RP))	**RP:** Clear and timely **Response**	**RP**: Actively agrees and integrates information/includes CareP in treatment
**Lucy**: *Here it comes*		
**Bettina looks at Clarisse:** *Yeah, now try the cologne on this side* ((points to the upper left)) *see if he'll look at it up there*	**RP:** Asks and proposes a plan of action.
**Clarisse** moves the cologne bottle into **Ken's** view and sprays the scent. He turns his eyes and looks towards Clarisse and it		**CareP**: Agrees/follows through with RP's plan of action/ask
**Bettina:** *Yeah, cool!* ((excited voice))		
**Clarisse looks at Bettina:** *He liked what he saw?*	**RP**: Assessment—Timely and transparent communication of what is seen	
**Bettina:** *Yeah, he was looking at it and his eyes went from this to there.*	**CareP**: asks for evaluation or offers evaluation	
	**RP**: Assessment—Clearly responds and states evaluation of action; announces; agrees; confirms decision	**RP:** Maintains eye contact; *Assesses* Pt's behaviour; acknowledges; agrees/confirms
		Micro‐decision **concludes**

Abbreviations: CareP = Clarisse; CSs = collaboration skills; IE = information exchange; Patient = Ken; RP = Bettina; Lucy.

##### Collaboration Skills

Relevant parties bring a variety of skills to clinical encounters. What constitutes CSs is an empirical question, that is, informed by how people orient to each other via talk and bodily conduct, and the demands of the environment in which they interact. Researchers use conceptual frameworks from behavioural sciences to discern these skills and environmental demands. What these frameworks have in common is a focus on communication skills as an essential part of clinical competencies and a critical element in effective collaboration [52, 53, 54]. Communication skills are necessary for collaboration but not sufficient on their own. We can observe whether and how people's behaviour is considerate, respectful, cooperative and so on. Verbal communication skills that may support engagement and collaboration might include word choice (e.g., framing decisions or actions as ‘we’ vs. ‘I’); an attitude of listening to understand; asking open‐ended questions or inviting each other to be part of the encounter. Nonverbal or paraverbal communication skills may include the direction of one's gaze, attunement to one's own and other's body language, gestures such as head nods to indicate listening and affirmation and voice tone. Behaviours that show disrespect or non‐collaboration might include use of dismissive language, curt responses, closed‐ended questions or physically turning away. The environmental demands in which people interact (such as duty to treat) are less well articulated. We are inspired by Kielhofner's model of human occupation [50] and Maynard's ethnographic and ethnomethodological research [55] to understand CSs in clinical settings. In the ‘mint or cologne’ example, the care partner provides timely and person‐centred information about the cologne and their loved one's preferences; the practitioners positively acknowledge this and integrate the information in clear and timely ways by asking the care partner to position the cologne and proposing a plan of action. In Table 3, we describe instances of IE and CSs from our example. We anticipate that as we analyse more data, we will refine and deepen our descriptions of these categories.

#### 5.2.2 Contextual

These dimensions are less easy to observe without analytic frameworks. They are the experiences, beliefs, values and meanings that relevant parties bring to a situation and inform their behaviours, including perceptions about the complexity and value of a decision, and the timing of the encounter vis‐a‐vis a patient's treatment trajectory overall and in a particular moment. Contextual factors influence behaviours in ways that may not always be obvious to participants of interactions since they are part of the experiential fabric of living in the world.

##### Experiences, Habits and Beliefs

Care partners' and practitioners' past and current experiences with each other and with healthcare settings, their beliefs about recovery and what they perceive and value as meaningful are relevant in the micro‐decision‐making environment. Through caregiving, care partners [2, 18] become knowledgeable about medical conditions and jargon, learn to interact with multiple healthcare services (insurance agencies, durable medical equipment companies), use medical equipment, administer assessments, become self‐advocates—in short, they experience a personal transformation and ‘wear a lot of hats’, as one care partner shared. They may also experience ambiguous loss [56, 57]. Practitioners are challenged to care for patients in DoC due to clinical uncertainty and may experience a lack of confidence [11]. We found [48] that both care partners and practitioners are challenged by the fluctuating quality of patients' behaviours and the limitations of discerning evidence of improvement (or lack thereof).

Care partners may perceive certain behaviours to have significance that may not be shared by practitioners. For instance, a care partner may place importance on the sound of a loved one's yawn because it is reminiscent of their loved one's pre‐injury behaviour. The yawn was a meaningful event in a care partner's day. For practitioners, a yawn is a vocalization that may or may not be perceived as significant or relevant. It may or may not have therapeutic salience. Practitioners may be inclined to perceive such behaviours as mere reflexes. Since people often make decisions based on their perceptions of what is meaningful, the decisions of practitioners and care partners may not align with each other [4].

##### Time Is a Complicated yet Essential Factor in RCSDM

Patients, care partners and practitioners interact with each other multiple times a day or week or month, which affects their rapport, expectations and collaboration skills. Practitioners at the end of a shift may find themselves less able to clearly articulate information, while care partners who have participated in several encounters that day may have drained their cognitive and emotional resources for decision‐making. For patients and their recovery outcomes, it matters whether they have been treated in rehabilitation for 2 weeks or 2 months, or whether an OT session, for example, occurred right after a strenuous PT session. Furthermore, those caring for loved ones over an extended time begin to speak in technical terms have facility with medical equipment and clinical procedures and may experience their own personal transformations as a result of this caring [57, 58]. The person's overall recovery trajectory [32] (e.g., primary hospitalization, rehabilitation episode or outpatient visit) and their ongoing life story are relevant when making decisions even though these dimensions may not be explicitly stated in an encounter.

##### Perceived Value and Meaning of Micro‐Decisions

Micro‐decisions are made within affective and ethical contexts that are often unstated, implicit and sometimes even unrecognized manifestations of feelings, duties and values. Patients, care partners and practitioners all bring these to the situation. Whether, when, where and how they enact those values and feelings is an empirical matter. For practitioners, a duty to treat is an ingrained ethic as is looking for signs that treatments are having the desired effect (as defined by disciplinary practice standards, insurance requirements and other ethical–legal obligations). For care partners, a duty to care may contextualize the ethical and affective ways they express love and support or a legally bound relationship (marriage, paid caregiving, among others). From within these affective and ethical perceptions, some micro‐decisions may be interpreted as more important than others, such as when a patient's yawn reminds a care partner of their pre‐injury loved one thus offering them a sign to remain hopeful.

### 5.3 Level 3: Roles and Relationships

There are multiple and intersecting roles and relationships that can be identified in a rehabilitation encounter. Simply put, interactions among patients, care partners and practitioners generate relationships that form the intersubjective environment within and from which all relevant parties draw to make decisions. Relationships have pasts that influence present interactions in encounters [59, 60]. As they interact with one another, care partners, practitioners and patients (even those in DoC) learn from each other about what each other values, and their communication styles may deepen engagement with each other or foster disengagement [61, 62, 63]. Interactions influence trust and rapport and support new understandings of each other's preferences and needs that may encourage adjusting expectations. Relationships are dynamic, and interactions among relevant parties are complex microsystems. It is outside the scope of this paper to describe all the psychological, communication and philosophical exigencies of relationships. We present five roles and relationships and their impact on micro‐decisions in rehabilitation encounters.

The five key relationships are care partner‐loved one, care partner‐care partner, care partner‐practitioner, practitioner‐practitioner (interprofessional) and practitioner‐patient. Relevant patient characteristics include medical conditions, cognitive capacity, demographics and other resources and support they may need (financial, social, legal, etc.).

1. Care partner‐loved one: This relationship may have a long history before the current caregiving experience or may be a continuation of the caregiving experience for children and young adults. There may have been a level of interdependency such as between life partners or siblings. Essential to SDM is inclusion of patient preferences and values; therefore, **the extent to which the care partner knows or believes they know the loved one's preferences and values is an important contribution to SDM**. This relationship itself may be influenced by the nature of the caregiving (e.g., full‐ or part‐time, intimate/all‐inclusive, casual), patient's advance directives, legal surrogacy and the type and quality of the relationship before this caregiving experience. For instance, when a care partner is a close relative rather than a paid attendant (who did not know the patient pre‐injury or illness), the relative may be better able to advocate on behalf of the person in ways congruent with their wishes.
2. Care partner‐care partner: Multiple care partners may be involved in a person's care. These care partners may be informal, familial, paid or a combination; they may engage with each other in more or less collaborative or amicable ways; there may be a primary care partner or a legal surrogate (who may or may not be the primary care partner); and these care partners may be proximal to each other or geographically separated. **The relationships among care partners before the current caregiving situation as well as with the loved one may affect ideas about what may count as reasonable goals for the loved one and what values and preferences would be most important to prioritize**. Relationships among care partners likely influence the primary care partner's ability to care for themselves as they care for their loved one which in turn influences their participation in SDM with practitioners.
3. Care partner‐practitioner: The literature states that practitioners may be concerned about how much involvement a family care partner should have in decision‐making. Practitioners may be concerned about border crossing—care partners challenging professional autonomy or expertise [57]. Even though practitioners may see care partners as important to a patient's clinical care, they may still see care partners as a means to a patient's clinical ends rather than as active partners in the decision‐making process. In other words, practitioners may treat care partners in a transactional rather than collaborative manner. Though educating care partners is an important aspect of person‐ and family‐centred rehabilitation, practitioners report concerns with care partners' emotional reactions to patients' responses [3] and may struggle with setting ‘realistic expectations’ for care partners while remaining hopeful when interacting with them [58]. Our prior studies indicate **care partners have both supportive and challenging interactions with rehabilitation practitioners** [4]. Care partners may feel heard and included but may also feel they are being dismissed. An important factor in some care partner‐practitioner relationships is that it is the care partners who can legally make decisions for the patient.
4. Practitioner‐practitioner (interprofessional): The literature on team effectiveness and interprofessional collaboration is vast and beyond the scope of this paper [3, 4, 58], but in sum, it suggests that relationships among healthcare practitioners influence their care practices [31, 64, 65, 66, 67]. We underscore that **how practitioners interact with each other is influenced by personal, professional, and organizational patterns of behaviours** including the team atmosphere on the unit/floor, the care model fostered at work and other local, cultural and experiential factors that practitioners bring with them or find at work. The interprofessional SDM (IP‐SDM) model [68] can be instructive here. While it has some of the same limitations related to PMR as other models of SDM, as described above, there is a specific focus on interprofessional care, teamwork and the need to train for interprofessional practice. In IP‐SDM, each practitioner has a role in supporting patients and families in understanding information, eliciting preferences and making decisions. Similar to our model, social forces are included as ‘environment’ and include social norms, organizational routines and institutional structure.
5. Practitioner‐patient: While persons with cognitive and communication disabilities that require high levels of support are not seen as competent to decide for themselves, nonetheless, they may show important interactional and conversational competencies [69], highlighting implicit collaborative practices and features of encounters. On an explicit level, practitioners use therapeutic activities to establish a relationship with the patient and to provide person‐centred care [4] such as ‘trying things’, thinking outside the box, improvising and innovating in their effort to care [10].

### 5.4 Level 4: Social Forces

We use the term ‘social forces’ to describe the intersubjective environment in which relationships and interactions take place. The demands and influences that environments exert are often hidden from our awareness. This level is perhaps best captured by the parable that opened David Foster Wallace's well‐known commencement speech ‘This is water’ [70].

There are these two young fish swimming along and they happen to meet an older fish swimming the other way, who nods at them and says, ‘Morning, boys. How's the water?’

And the two young fish swim on for a bit, and then eventually one of them looks over at the other and goes, ‘What the hell is water?’

In saying that these forces are like water, we mean they are ubiquitous, omnipresent, often unspoken and hard to be aware of. Social scientists have captured this level through concepts such as habitus, ideology, culture and so forth to point to how subtly (or not so subtly) they affect what we do, how we do what we do and who we understand ourselves to be. It requires practice to take notice [71] of how these influences are part of our norms, expectations, assumptions and the ways we organize our economy, educational and legal systems. For instance, unlike other nations, in the United States, much of the healthcare system is based on a for‐profit, free‐market economic system with its particular instantiations of ableism, racism, sexism and other ‐isms that epigenetically and intersectionally influence our actions at both individual and community levels. Most often, we are not aware of these ‐isms in our behaviours or in the environments we inhabit; they are the water we swim in. How these social forces are present and influence rehabilitation encounters is an empirical question we will explore in our future work. In our example, some social forces that influence the RPs actions may be grounded in professional, institutional and disciplinary expectations and requirements and ethical duties to help patients improve even when they are not responding, to provide care based on what is necessary for (insurance) billing purposes and to demonstrate evidence of patients' progress. The care partners may be influenced by social forces such as epistemic injustice where their experiences with their loved ones may not be solicited, received or listened to as they may not align with the rehabilitation canon [11]. Power dynamics exist within healthcare settings. In our example, we note their fluidity when, at first, the RPs reject (with an apology) the care partner's first offer to use cranberry juice rather than the canonical mint. In the same breath, the RP articulated the ubiquitous reality of clinical uncertainty when treating persons in DoC and their tinkering skills [11] (‘we are just trying anything and everything’). This uncertainty is a place of possible doubt *and* openness to try doing something differently, that is, to tinker. The RPs respond with openness to tinker the second time the care partner initiates and they incorporate the cologne.

## 6 Conclusion

In this paper, we proposed a new process model for relationship‐centred SDM that is relevant to PMR settings. The RCSDM is the result of literature reviews, data analyses, discussions and engagement with SDM experts, rehabilitation professionals and care partners. Our model focuses on micro‐decisions during rehabilitation encounters to unpack how decisions are made. We explicate the interactional and contextual dimensions of decisions that are grounded in the multiple and intersecting relationships among relevant parties.

## Author Contributions

**Christina Papadimitriou:** conceptualization, methodology, funding acquisition, writing–original draft, writing–review and editing, visualization, supervision, resources, formal analysis. **Marla L. Clayman:** conceptualization, methodology, visualization, writing–original draft, writing–review and editing, formal analysis. **Trudy Mallinson:** conceptualization, funding acquisition, writing–original draft, writing–review and editing, visualization, methodology, formal analysis. **Jennifer A. Weaver:** data curation, investigation, writing–original draft, writing–review and editing, conceptualization, methodology, visualization, formal analysis. **Ann Guernon:** conceptualization, methodology, visualization, writing–review and editing, writing–original draft, formal analysis. **Albert J. Meehan:** methodology, visualization, writing–review and editing, formal analysis, conceptualization. **Trisha Kot:** investigation, writing–original draft, writing–review and editing, visualization. **Paige Ford:** investigation, writing–original draft, writing–review and editing, visualization. **Roger Ideishi:** writing–review and editing; writing–original draft. **Christina Prather:** writing–review and editing, visualization, writing–original draft. **Philip van der Wees:** writing–review and editing, writing–original draft.

## Ethics Statement

This study was conducted in accordance with the ethical principles outlined in the Declaration of Helsinki. Approval for the study was obtained from the Institutional Review Board (IRB) of the George Washington University (Approval Number: NCR191275, title of study: Understanding how rehabilitation practitioners select and use assessments; PI: Jennifer A. Weaver). All participants provided informed consent before their participation in the study.

## Conflicts of Interest

The authors declare no conflicts of interest.

## Data Availability

The data that support the findings of this study are available from the corresponding author upon reasonable request.

## References

[1] C. Papadimitriou , T. Mallinson , J. Weaver , et al., “Proposing a Relationship‐Centered SDM Process Conceptual Model for Chronic Disability,” Patient Education and Counseling 109 (2023): 11, 10.1016/j.pec.2022.10.035.

[2] T. Pape , J. Weaver , C. Papadimitriou , et al., “No One Listens to Me: Working With Caregivers to Listen to Their Caring Experiences,” Archives of Physical Medicine and Rehabilitation 100, no. 10 (2019): e102–e103, 10.1016/j.apmr.2019.08.296.

[3] C. Papadimitriou , J. Weaver , A. Guernon , et al., “Caregiver Perceptions of Their Communication With Rehabilitation Providers Who Treat Persons in Disordered States of Consciousness,” Archives of Physical Medicine and Rehabilitation 103, no. 3 (2022): e18, 10.1016/j.apmr.2022.01.048.

[4] A. Guernon , J. Weaver , T. Kot , et al., “‘My Person’ or ‘A Person’: CarePartner and Practitioner Perceptions Caring for Persons in Disordered Consciousness,” Archives of Physical Medicine and Rehabilitation 103, no. 12 (2022): e80–e81, 10.1016/j.apmr.2022.08.639.

[5] F. Légaré and H. O. Witteman , “Shared Decision Making: Examining Key Elements and Barriers to Adoption Into Routine Clinical Practice,” Health Affairs 32, no. 2 (2013): 276–284, 10.1377/hlthaff.2012.1078. 23381520

[6] H. Bomhof‐Roordink , F. R. Gärtner , A. M. Stiggelbout , and A. H. Pieterse , “Key Components of Shared Decision Making Models: A Systematic Review,” BMJ Open 9, no. 12 (2019): e031763, 10.1136/bmjopen-2019-031763. PMC6937101 31852700

[7] F. R. Gärtner , H. Bomhof‐Roordink , I. P. Smith , I. Scholl , A. M. Stiggelbout , and A. H. Pieterse , “The Quality of Instruments to Assess the Process of Shared Decision Making: A Systematic Review,” PLoS One 13, no. 2 (2018): e0191747, 10.1371/journal.pone.0191747. 29447193 PMC5813932

[8] A. Rose , S. Rosewilliam , and A. Soundy , “Shared Decision Making Within Goal Setting in Rehabilitation Settings: A Systematic Review,” Patient Education and Counseling 100, no. 1 (2017): 65–75, 10.1016/j.pec.2016.07.030. 27486052

[9] L. R. Beach , “Broadening the Definition of Decision Making: The Role of Prechoice Screening of Options,” Psychological Science 4, no. 4 (1993): 215–220.

[10] D. Stacey , F. Légaré , S. Pouliot , J. Kryworuchko , and S. Dunn , “Shared Decision Making Models to Inform an Interprofessional Perspective on Decision Making: A Theory Analysis,” Patient Education and Counseling 80, no. 2 (2010): 164–172, 10.1016/j.pec.2009.10.015. 19945813

[11] C. Papadimitriou , J. A. Weaver , A. Guernon , E. Walsh , T. Mallinson , and T. L. B. Pape , “Fluctuation Is the Norm: Rehabilitation Practitioner Perspectives on Ambiguity and Uncertainty in Their Work With Persons in Disordered States of Consciousness After Traumatic Brain Injury,” PLoS One 17 4 (2022): e0267194, 10.1371/journal.pone.0267194. 35446897 PMC9022828

[12] C. Gómez‐Vírseda , Y. De Maeseneer , and C. Gastmans , “Relational Autonomy: What Does It Mean and How Is It Used in End‐of‐Life Care? A Systematic Review of Argument‐Based Ethics Literature,” BMC Medical Ethics 20, no. 1 (2019): 76, 10.1186/s12910-019-0417-3. 31655573 PMC6815421

[13] A. Ho , “Relational Autonomy or Undue Pressure? Family's Role in Medical Decision‐Making,” Scandinavian Journal of Caring Sciences 22, no. 1 (2008): 128–135, 10.1111/j.1471-6712.2007.00561.x. 18269432

[14] C. Mackenzie and N. Stoljar , Relational Autonomy: Feminist Perspectives on Autonomy, Agency, and the Social Self (Oxford, UK: Oxford University Press, 2000).

[15] C. Mackenzie , “The Importance of Relational Autonomy and Capabilities for an Ethics of Vulnerability,” in Vulnerability, eds. C. Mackenzie , W. Rogers , and S. Dodds (Oxford, UK: Oxford University Press, 2013), 33–59, 10.1093/acprof:oso/9780199316649.003.0002.

[16] C. Ells , M. R. Hunt , and J. Chambers‐Evans , “Relational Autonomy as an Essential Component of Patient‐Centered Care,” IJFAB: International Journal of Feminist Approaches to Bioethics 4, no. 2 (2011): 79–101, 10.3138/ijfab.4.2.79.

[17] T. W. Bickmore , S. E. Mitchell , B. W. Jack , M. K. Paasche‐Orlow , L. M. Pfeifer , and J. O'Donnell , “Response to a Relational Agent by Hospital Patients With Depressive Symptoms,” Interacting With Computers 22, no. 4 (2010): 289–298, 10.1016/j.intcom.2009.12.001. 20628581 PMC2901553

[18] J. Weaver , C. Papadimitriou , L. Davidson , A. Guernon , P. Van Der Wees , and T. Mallinson , “Explicating Integrative and Declarative Shared Treatment Decision Making in Rehabilitation Encounters When Patients Are Without a Voice: A Grounded Theory Analysis. Presented at: ACRM 98th Annual Conference,” Archives of Physical Medicine and Rehabilitation 103, no. 3 (2021): e22, 10.1016/j.apmr.2022.01.060.

[19] J. Craft Weaver , Translating Assessments Into Practice Using Principles of Patient‐Centered Measurement: An Exemplar Using the Coma Recovery Scale‐Revised (Washington: The George Washington University, 2021), 10.4079/THS2021.02.

[20] J. Weaver , T. Mallinson , L. Davidson , C. Papadimitriou , A. Guernon , and P. Der Wees , “Exploring Shared Decision Making Between Family Caregivers, Persons With Disorders of Consciousness, and Rehabilitation Therapists in Acute Care,” supplement, The American Journal of Occupational Therapy 75, no. S2 (2021): 7512510247p1, 10.5014/ajot.2021.75S2-RP247.

[21] J. Hamann and S. Heres , “Why and How Family Caregivers Should Participate in Shared Decision Making in Mental Health,” Psychiatric Services 70, no. 5 (2019): 418–421, 10.1176/appi.ps.201800362. 30784381

[22] R. L. Daly , F. Bunn , and C. Goodman , “Shared Decision‐Making for People Living With Dementia in Extended Care Settings: A Systematic Review,” BMJ Open 8, no. 6 (2018): e018977, 10.1136/bmjopen-2017-018977. PMC6009462 29886439

[23] J. R. Covvey , K. M. Kamal , E. E. Gorse , et al., “Barriers and Facilitators to Shared Decision‐Making in Oncology: A Systematic Review of the Literature,” Supportive Care in Cancer 27, no. 5 (2019): 1613–1637, 10.1007/s00520-019-04675-7. 30737578

[24] J. D. Caputo , “Husserl, Heidegger and the Question of a “Hermeneutic” Phenomenology,” Husserl Studies 1, no. 1 (1984): 157–178, 10.1007/BF01569213.

[25] C. Papadimitriou , M. Clayman , and T. Mallinson , et al., “What Does Shared Decision Making Look Like in Chronic Disability Care? Adapting a Conceptual Process Model of SDM to Better Serve PM&R,” Archives of Physical Medicine and Rehabilitation 101, no. 12 (2020): e155, 10.1016/j.apmr.2020.10.090.

[26] E. H. Ofstad , J. C. Frich , E. Schei , R. M. Frankel , and P. Gulbrandsen , “Temporal Characteristics of Decisions in Hospital Encounters: A Threshold for Shared Decision Making? A Qualitative Study,” Patient Education and Counseling 97, no. 2 (2014): 216–222, 10.1016/j.pec.2014.08.005. 25176608

[27] M. A. Smith , M. L. Clayman , J. Frader , et al., “A Descriptive Study of Decision‐Making Conversations During Pediatric Intensive Care Unit Family Conferences,” Journal of Palliative Medicine 21, no. 9 (2018): 1290–1299, 10.1089/jpm.2017.0528. 29920145

[28] A. Michalsen , A. C. Long , F. DeKeyser Ganz , et al., “Interprofessional Shared Decision‐Making in the ICU: A Systematic Review and Recommendations From an Expert Panel,” Critical Care Medicine 47, no. 9 (2019): 1258–1266, 10.1097/CCM.0000000000003870. 31169620

[29] R. C. Laidsaar‐Powell , P. N. Butow , S. Bu , et al., “Physician–Patient–Companion Communication and Decision‐Making: A Systematic Review of Triadic Medical Consultations,” Patient Education and Counseling 91, no. 1 (2013): 3–13, 10.1016/j.pec.2012.11.007. 23332193

[30] C. De Haas , J. Grace , J. Hope , and M. Nind , “Doing Research Inclusively: Understanding What It Means to do Research With and Alongside People With Profound Intellectual Disabilities,” Social Sciences 11, no. 4 (2022): 159, 10.3390/socsci11040159.

[31] A. Opie , Thinking Teams/Thinking Clients: Knowledge‐Based Team Work (New York: Columbia University Press, 2001).

[32] M. L. Clayman , P. Gulbrandsen , and M. A. Morris , “A Patient in the Clinic; a Person in the World. Why Shared Decision Making Needs to Center on The Person Rather Than the Medical Encounter,” Patient Education and Counseling 100, no. 3 (2017): 600–604, 10.1016/j.pec.2016.10.016. 27780646

[33] A. Peterson , K. M. Kostick , K. A. O'Brien , and J. Blumenthal‐Barby , “Seeing Minds in Patients With Disorders of Consciousness,” Brain Injury 34, no. 3 (2020): 390–398, 10.1080/02699052.2019.1706000. 31880960

[34] J. Weaver , C. Mueller , A. McGuire , et al., “Translating the Coma Recovery Scale‐Revised Into Clinical Practice Using Person‐Centered Measurement Principles,” Archives of Physical Medicine and Rehabilitation 103, no. 12 (2022): e68, 10.1016/j.apmr.2022.08.604.

[35] G. Elwyn , D. Frosch , R. Thomson , et al., “Shared Decision Making: A Model for Clinical Practice,” Journal of General Internal Medicine 27, no. 10 (2012): 1361–1367, 10.1007/s11606-012-2077-6. 22618581 PMC3445676

[36] M. M. W. Karlsen , M. B. Happ , A. Finset , K. Heggdal , and L. G. Heyn , “Patient Involvement in Micro‐Decisions in Intensive Care,” Patient Education and Counseling 103, no. 11 (2020): 2252–2259, 10.1016/j.pec.2020.04.020. 32493611

[37] C. Cott , “Client‐Centred Rehabilitation: Client Perspectives,” Disability and Rehabilitation 26, no. 24 (2004): 1411–1422, 10.1080/09638280400000237. 15764361

[38] T. S. Jesus , F. A. Bright , C. S. Pinho , C. Papadimitriou , N. M. Kayes , and C. A. Cott , “Scoping Review of the Person‐Centered Literature in Adult Physical Rehabilitation,” Disability and Rehabilitation 43, no. 11 (2021): 1626–1636, 10.1080/09638288.2019.1668483. 31553633

[39] C. Papadimitriou and C. Cott , “Client‐Centred Practices and Work in Inpatient Rehabilitation Teams: Results From Four Case Studies,” Disability and Rehabilitation 37, no. 13 (2015): 1135–1143, 10.3109/09638288.2014.955138. 25163833

[40] P. Berne , Skin, Tooth, and Bone—The Basis of Movement Is Our People: A Disability Justice Primer, 2nd ed. (San Francisco: Sins Invalid, 2017), https://www.sinsinvalid.org/disability-justice-primer. 10.1080/09688080.2017.1335999 28784067

[41] L. I. Iezzoni , M. M. McKee , M. A. Meade , M. A. Morris , and E. Pendo , “Have Almost Fifty Years of Disability Civil Rights Laws Achieved Equitable Care?: Overview Examines 50 Years of US Disability Civil Rights Laws,” Health Affairs 41, no. 10 (2022): 1371–1378, 10.1377/hlthaff.2022.00413. 36190880 PMC10359967

[42] P. Kontos , K. L. Miller , and A. P. Kontos , “Relational Citizenship: Supporting Embodied Selfhood and Relationality in Dementia Care,” Sociology of Health & Illness 39, no. 2 (2017): 182–198, 10.1111/1467-9566.12453. 28177149

[43] G. Mitchell , S. L. Dupuis , P. Kontos , C. Jonas‐Simpson , and J. Gray , “Disrupting Dehumanising and Intersecting Patterns of Modernity With a Relational Ethic of Caring,” International Practice Development Journal 10, no. 1 (2020): 1–15, 10.19043/ipdj.101.002.

[44] E. Gendlin , Experiencing and the Creation of Meaning: A Philosophical and Psychological Approach to the Subjective, 1st ed. (Evanston: Northwestern University Press, 1997).

[45] L. Preston , “The Edge of Awareness: Gendlin's Contribution to Explorations of Implicit Experience,” International Journal of Psychoanalytic Self Psychology 3, no. 4 (2008): 347–369, 10.1080/15551020802337419.

[46] M. van Manen , Researching Lived Experience: Human Science for an Action Sensitive Pedagogy, 2nd ed. (New York: Routledge, 2018).

[47] N. J. Enfield , How We Talk: The Inner Workings of Conversation (New York: Basic Books, 2017).

[48] P. J. Hutchison , K. McLaughlin , T. Corbridge , et al., “Dimensions and Role‐Specific Mediators of Surrogate Trust in the ICU,” Critical Care Medicine 44, no. 12 (2016): 2208–2214, 10.1097/CCM.0000000000001957. 27513360 PMC5219932

[49] K. Forsyth , J. S. Lai , and G. Kielhofner , “The Assessment of Communication and Interaction Skills (ACIS): Measurement Properties,” British Journal of Occupational Therapy 62, no. 2 (1999): 69–74, 10.1177/030802269906200208.

[50] G. Kielhofner , Model of Human Occupation: Theory and Application, 4th ed. (Philadelphia: Lippincott Williams & Wilkins, 2008).

[51] J. Heritage , “Epistemics in Conversation,” in The Handbook of Conversation Analysis, 1st ed., eds. J. Sidnell and T. Stivers (New York: Wiley, 2012), 370–394, 10.1002/9781118325001.ch18.

[52] A. Roman and A. V. Pineiro , “Nursing Communication Skills Training: Added Importance During Crises,” Journal for Nurses in Professional Development 39, no. 4 (2023): E86–E92, 10.1097/NND.0000000000000868. 35175999

[53] E. Yazdanparast , A. Arasteh , S. Ghorbani , and M. Davoudi , “The Effectiveness of Communication Skills Training on Nurses' Skills and Participation in the Breaking Bad News,” Iranian Journal of Nursing and Midwifery Research 26, no. 4 (2021): 337, 10.4103/ijnmr.IJNMR_150_20. 34422614 PMC8344626

[54] E. Khodadadi , H. Ebrahimi , S. Moghaddasian , and J. Babapour , “The Effect of Communication Skills Training on Quality of Care, Self‐Efficacy, Job Satisfaction and Communication Skills Rate of Nurses in Hospitals of Tabriz, Iran,” Journal of Caring Science 2 (2013): 27–37, 10.5681/JCS.2013.004. PMC4161104 25276707

[55] D. W. Maynard and J. Turowetz , Autistic Intelligence: Interaction, Individuality, and the Challenges of Diagnosis (Chicago: University of Chicago Press, 2022).

[56] H. Carel , Phenomenology of Illness (Oxford, UK: Oxford University Press, 2016).

[57] C. Mattingly , The Paradox of Hope: Journeys Through a Clinical Borderland (Oakland: University of California Press, 2010).

[58] J. Weaver , A. Guernon , I. Abuzahra , et al., “Exploring Family Care Partners' and OTs' Hope When Caring for a Person With Disordered Consciousness Resulting From a Brain Injury,” supplement, American Journal of Occupational Therapy 76, no. S1 (2022): 7610510021p1, 10.5014/ajot.2022.76S1-RP21.

[59] D. A. Stone and C. Papadimitriou , “Exploring Heidegger's Ecstatic Temporality in the Context of Embodied Breakdown,” Schutzian Research 2 (2010): 137–154.

[60] A. C. Sparkes , “‘The Second I Got the Phone Call, Everything Changed.’ Exploring the Temporal Experiences of the Spouses and Partners of Spinal Cord Injured Sportsmen,” Qualitative Research in Sport 15 (2022): 1–19.

[61] S. D. Ronis , L. C. Kleinman , and K. C. Stange , “A Learning Loop Model of Collaborative Decision‐Making in Chronic Illness,” Academic Pediatrics 19, no. 5 (2019): 497–503, 10.1016/j.acap.2019.04.006. 31009759 PMC8127066

[62] Deborah Tannen , “Interactional Sociolinguistics as a Resource for Intercultural Pragmatics,” Intercultural Pragmatics 2 (2005): 205–208.

[63] D. Tannen , Conversational Style: Analyzing Talk Among Friends (Oxford, UK: Oxford University Press, 2005).

[64] G. D. Heinemann and A. M. Zeiss , Team Performance in Health Care: Assessment and Development, 2nd ed. (Berlin, Germany: Springer, 2002).

[65] J. H. Gittell , M. Godfrey , and J. Thistlethwaite , “Interprofessional Collaborative Practice and Relational Coordination: Improving Healthcare Through Relationships,” Journal of Interprofessional Care 27, no. 3 (2013): 210–213, 10.3109/13561820.2012.730564. 23082769

[66] J. Gittell , “Coordinating Work Through Relationships of Shared Goals, Shared Knowledge and Mutual Respect,” in Relational Perspectives in Organizational Studies: A Research Companion, eds. O. Kyriakidou and M. F. Özbilgin (Cheltenham, UK: Edward Elgar Publishing, 2006), 74–94.

[67] D. S. Havens , J. Vasey , J. H. Gittell , and W. T. Lin , “Relational Coordination Among Nurses and Other Providers: Impact on the Quality of Patient Care: Relational Coordination Among Nurses and Other Providers,” Journal of Nursing Management 18, no. 8 (2010): 926–937, 10.1111/j.1365-2834.2010.01138.x. 21073566

[68] F. Légaré , D. Stacey , S. Gagnon , et al., “Validating a Conceptual Model for an Inter‐Professional Approach to Shared Decision Making: A Mixed Methods Study,” Journal of Evaluation in Clinical Practice 17, no. 4 (2011): 554–564, 10.1111/j.1365-2753.2010.01515.x. 20695950 PMC3170704

[69] C. Papadimitriou , L. Lindemann , and A. J. Meehan , “Making the Visible Seen: The Interactional Competence of a Person in a Disordered State of Consciousness,” Social Science & Medicine 336 (2023): 116261, 10.1016/j.socscimed.2023.116261. 37806147

[70] “This is Water by David Foster Wallace,” Farnam Street, 2012, https://fs.blog/david-foster-wallace-this-is-water/.

[71] R. C. Scharff and D. A. Stone , “Transdisciplinarity Without Method: On Being Interdisciplinary in a Technoscientific World,” Human Studies 45, no. 1 (2022): 1–25, 10.1007/s10746-021-09616-0.

